# Acetylcholinesterase Inhibitory and Antioxidant Activity of the Compounds Isolated from *Vanda roxburghii*

**DOI:** 10.1155/2021/5569054

**Published:** 2021-03-27

**Authors:** Salim Ahammed, Rejina Afrin, Nasim Uddin, Yusuf Al-Amin, Kamrul Hasan, Uzzal Haque, K. M. Monirul Islam, A. H. M. K. Alam, Toshihisa Tanaka, Golam Sadik

**Affiliations:** ^1^Department of Pharmacy, University of Rajshahi, Rajshahi 6205, Bangladesh; ^2^Department of Pharmacy, East West University, Dhaka, Bangladesh; ^3^Department of Psychiatry, Osaka University Graduate School of Medicine, Osaka, Japan

## Abstract

*Vanda roxburghii* has been used in traditional medicine to treat nervous system disorders including Alzheimer's disease (AD). We reported earlier a high acetylcholinesterase inhibitory and antioxidant activity in the chloroform fraction of this plant. Therefore, this study was designed to explore the compounds with acetylcholinesterase inhibitory and antioxidant activities from the chloroform fraction of *Vanda roxburghii*. Phytochemical investigation led to the isolation for the first time of a fatty acid ester: methyl linoleate (1), and three phenolics: syringaldehyde (2), vanillin (3), and dihydroconiferyl dihydro-*p*-coumarate (4) along with the previously reported compound gigantol (5). Among the isolates, vanillin (3) and dihydroconiferyl dihydro-*p*-coumarate (4) were found to significantly inhibit the activity of acetylcholinesterase, scavenge the free radicals, exhibit the reducing power and total antioxidant activity, and effectively reduce the peroxidation of lipid. Gigantol (5) and syringaldehyde (2), despite lacking the activity against acetylcholinesterase, exhibited antioxidant activity. Among the compounds, gigantol (5) appeared to be the most potent antioxidant. These findings revealed that *V. roxburghii* contained compounds with potential acetylcholinesterase inhibitory and antioxidant activity, which support its traditional use in the treatment of AD.

## 1. Introduction

AD is an age-related neurodegenerative disorder of the elderly people with symptoms of memory loss and cognition. Cholinergic deficit, deposition of amyloid plaque and formation of neurofibrillary tangles, oxidative stress, and inflammation are the prominent features of AD [1, 2]. Although several mechanisms of AD pathogenesis have been delineated, acetylcholinesterase (AChE) still remains as the most attractive therapeutic target for symptomatic improvement of AD. Inhibition of AChE elevates the level of acetylcholine in the synaptic cleft and improves the cholinergic neurotransmission [3, 4]. To date, only four drugs have been approved by the FDA for treating AD, among which three are AChE inhibitors—donepezil, galantamine, and rivastigmine. Although these drugs are effective in ameliorating the memory and cognition, they cannot stop the disease progression [5]. Mounting evidences suggest that oxidative stress is also involved in the progression of AD [6, 7]. It has been clearly demonstrated that Abeta protein, the major constituent of senile plaque, can increase the free radical production and reactive oxygen species (ROS), leading to oxidative stress [8]. ROS can attack most of the cell constituents including DNA, protein, and lipid, and an increased oxidation of DNA and protein and peroxidation of lipid are observed in the brain with AD [9]. Antioxidants have been found to reduce the oxidative stress-induced damage in AD [10]. Therefore, there is growing consensus that an agent that can modulate the multiple pathologic processes may yield a better therapeutic effect. In recent times, interest in medicinal plants for natural drug has increased significantly due to toxicity of the synthetic drugs.

*Vanda roxburghii*, locally known as Rasna, is an epiphyte of the Orchidaceae family and typically found all over Bangladesh. In Ayurveda and Unani, the traditional medical systems, the plant has been indicated for treatment of different ailments [11]. In Unani, the plant has been used as tonic to the brain and to treat diseases of the nervous system including AD. The plant is also useful for the treatment of pain, inflammation, rheumatoid arthritis, sciatica, liver disease, bronchitis, hiccough, piles, and fever. The root of the plant is reported to have activity against bacterial infection and tuberculosis, diseases of the abdomen, tremor, and otitis [12]. Phytochemical analyses revealed that the plant contains a large amount of phenolics and flavonoids [13]. Biological investigation of the plant reported the wound healing, anti-arthritic, and anti-nociceptive activities [14–16]. In a previous investigation, we reported the acetylcholinesterase inhibitory and antioxidant activities of the crude methanolic extract of this plant [13]. The IC50 value of the extract was 828.27 *μ*g/ml for acetylcholinesterase inhibition and 19.23 *μ*g/ml for DPPH scavenging activity. Further evaluation of the different fractions of the extract revealed the highest activity in the chloroform fraction with IC50 value of 221.13 *μ*g/ml for acetylcholinesterase inhibition and 5.76 *μ*g/ml for DPPH scavenging activity. Only a phenolic compound gigantol was isolated from the chloroform fraction, but its biological potential is yet to be completely delineated. Therefore, further study was carried out on the chloroform fraction of *V. roxburghii* to obtain an insight into the chemical compounds that could be contributing to the bioactivities.

## 2. Materials and Methods

### 2.1. Experimental Animals

Swiss albino mice of 6–8 months old were obtained from our own animal facility and used in this experiment for collection of brain to prepare AChE enzyme. International ethical guidelines were followed for dealing with the animals, and the procedures were approved by the Institutional Animal, Medical Ethics, Biosafety and Biosecurity Committee (IAMEBBC), Institute of Biological Sciences, University of Rajshahi, Bangladesh (Ethical approval no. 104).

### 2.2. Plant Collection

The root of *V. roxburghii* was collected from the Rajshahi University campus, Rajshahi, Bangladesh. It was identified and a voucher specimen (No. PH12) has been deposited at the herbarium of the Department of Botany, Rajshahi University, Bangladesh.

### 2.3. Extraction and Isolation of the Constituents

The dried powder of *V. roxburghii* (root, 1 kg) was exhaustively extracted with methanol by the cold extraction method to give 26.25 g of dry extract. It was then partitioned with chloroform, after defatting with petroleum ether, to obtain the chloroform fraction (7.54 g). The fraction was chromatographed in an open column packed with silica gel and eluted with n-hexane, dichloromethane, and methanol in increasing polarity. Based on similar TLC profile, the column fractions were combined that yielded five major portions, P1 (1.68 g), P2 (1.21 g), P3 (2.00 g), P4 (1.55 g), and P5 (2.51 g). P1, P2, and P3 were further purified by preparative TLC on silica gel with solvent system I to obtain the pure compounds 1 (25 mg), 2 (31 mg), and 3 (35 mg), respectively. P4 and P5 were similarly purified by preparative TLC on silica gel using solvent system II to get the pure compounds 4 (19 mg) and 5 (41 mg), respectively.

### 2.4. Characterization of the Compounds

The ^1^H and ^13^C-NMR spectra of the compounds 1–5 were acquired in CDCl_3_ by using Jeol-Ex 400 MHz and FT-NMR 100 MHz spectrometers. All the compounds were identified by comparing their spectral data with the published values in the literature [13, 17–19].

#### 2.4.1. Methyl Linoleate (1)

^1^H-NMR (CDCl_3_, *J* in Hz): *δ*5.31-5.37 (m, 4H, H-9, H-10, H-12, H-13), 3.65 ( s, 3H, C1-OCH_3_), 2.75 (t, 2H, 6.5, H-11), 2.29 (t, 2H, 7.5, H-2), 2.00-2.05 (m, 4H, H-8, H-14), 1.58-1.60 (m, 2H, H-3), 1.24-1.34 (m, 14H, H-4, H-5, H-6, H-7, H-15, H-16, H-17), 0.87 (t, 3H, 6.5, H-18). ^13^C-NMR (CDCl_3_): *δ* 174.30 (C-1), 130.24 (C-13), 130.07 (C-9), 128.30 (C-12), 128.06 (C-10), 51.46 (C1-OCH_3_), 34.12 (C-2), 31.54 (C-16), 29.71 (C-7), 29.60 (C-6), 29.36 (C-15), 29.17 (C-5), 29.13 (C-4), 29.11 (C-14), 27.21 (C-8), 25.64 (C-11), 25.54 (C-3), 22.59 (C-17), 14.28 (C-18).

#### 2.4.2. Syringaldehyde (2)

^1^H-NMR (CDCl_3_, *J* in Hz): *δ*9.84 (s, 1H, CHO), 7.13 (s, 2H, H-2, H-6), 6.50 (s, 1H, C4-OH), 3.94 (s, 6H, C3,5-OCH_3_).

#### 2.4.3. Vanillin (3)

^1^H-NMR (CDCl_3_, *J* in Hz): *δ*9.80 (s, 1H, CHO), 7.43 (d, 1H, 8.5, H-6), 7.42 (s, 1H, H-2), 7.02 (d, 1H, 8.5, H-5), 5.43 (s, 1H, C4-OH), 3.94 (s, 3H, C3-OCH_3_). ^13^C-NMR (CDCl_3_): 190.88 (CHO), 151.75 (C-4), 147.21 (C-3), 129.91 (C-1), 127.5 (C-6), 114.39 (C-5), 108.85 (C-2), 56.13 (C3-OCH_3_).

#### 2.4.4. Dihydroxyconiferyl Dihydro-*p*-Coumarate (4)

^1^H-NMR (CDCl_3_, *J* in Hz): *δ*7.03 (d, 2H, 8.6, H-2',H-6'), 6.82 (d, 1H, 8.5, H-5), 6.74 (d, 2H, 8.0, H-3', H-5'), 6.62 (brs, 1H, H-2); 6.58 (dd, 1H, 8.8, 1.8, H-6); 5.42 (s, 1H, C4-OH); 4.07 (t, 2H, 6.0, H-9), 3.93 (s, 3H, C3-OCH_3_); 2.86 (m, 2H, H-7'); 2.58 (m, 2H, H-8'); 2.56 (t, 2H, 8.0, H-7), *δ*1.9 (m, 2H, H-8). ^13^C-NMR (CDCl_3_): *δ*173.18 (C-9'), 154.31 (C-4'), 146.49 (C-3), 143.82 (C-4), 133.08 (C-1), 132.45 (C-1'), 129.38 (C-2'), 128.37 (C-6'), 120.94 (C-6), 115.37 (C-3'), 114.98 (C-5'), 114.37 (C-5), 110.96 (C-2), 63.87 (C-9), 55.88 (C3-OCH_3_), 36.30 (C-8'), 31.81 (C-7), 30.47 (C-8), 30.12 (C-7').

#### 2.4.5. Gigantol (5)

^1^H-NMR (CDCl_3_, *J* in Hz): *δ* 6.81 (d, 1H, 7.8, H-5”), 6.68 (d, 1H, 7.8, H-6”), 6.62(s, 1H, H-2”), 6.24 (brs, 2H, H-2', H-4'), 6.29 (s, 1H, H-6'), 3.82 (s, 3H, C5'-OCH_3_), 3.77 (s, 3H, C3”-OCH_3_), 2.82 (m, 4H, H-1, H-2). ^13^C-NMR (CDCl_3_): *δ* 160.85 (C-5´), 156.68 (C-3´), 146.27 (C-3”), 144.53 (C-1'), 143.81(C-4”), 133.65 (C-1”), 121.00 (C-6”), 114.29 (C-2”), 111.17 (C-5”), 108.06 (C-2'), 106.77 (C-6'), 99.03 (C-4'), 55.89 (C5'-OCH_3_), 55.27, (C3”-OCH_3_), 38.30 (C-2), 37.29 (C-1).

### 2.5. Assessment of AChE Inhibitory Activities

The slightly modified Ellman method (1961) was used for investigating the AChE inhibitory activity of the compounds using acetylthiocholine as substrate [20]. The AChE enzyme was prepared from mouse brain as described earlier [13]. Compound at different concentrations was mixed with AChE solution and incubated at 37°C for 15 min. The rates of hydrolysis by AChE was monitored by spectrophotometer at 405 nm immediately after adding an Ellman's reaction mixture containing 0.5 mM acetylthiocholine and 1 mM 5, 5′-dithio-bis (2-nitro benzoic acid) (DTNB) in a 50 mM phosphate buffer (pH 8.0) to the above reaction mixture. Donepezil was used as the standard AChE inhibitor. The percentage inhibition of AChE activity was calculated using the following formula:

(1) $$\begin{array}{c} \left[\frac{\left(\text{Absorbance of control}-\text{Absorbance of sample}\right)}{\text{Absorbance of control}}\right]\times 100\text{.} \end{array}$$

IC_50_ value could be calculated from the inhibition curve obtained by plotting the percent inhibition values against test concentrations of each compound.

Quality of assay was assessed by using *Z*' factor as follows [21]:

(2) $$\begin{array}{c} Z\prime =1-\frac{3\left(\sigma_{c+}+\sigma_{c-}\right)}{\left|\mu_{c+}-\mu_{c-}\right|}, \end{array}$$

where *σ*_*c*+_ and *σ*_*c*−_are the standard deviations of the positive and negative control and *μ*_*c*+_ and *μ*_*c*−_are the means of the positive and negative control, respectively.

Similarly, for evaluation of the performance of a compound, *Z* factor of compound was calculated by the following formula:

(3) $$\begin{array}{c} Z=1-\frac{3\left(\sigma_{s}+\sigma_{c}\right)}{\left|\mu_{s}-\mu_{c}\right|}, \end{array}$$

where *σ*_*s*_ and *σ*_*c*_ are the standard deviations of the sample and control and *μ*_*s*_ and *μ*_*c*_ are the means of the sample and control, respectively.

### 2.6. Assessment of Antioxidant Activity

#### 2.6.1. DPPH Radical Scavenging Assay

The DPPH radical scavenging ability of the compounds was estimated using the method described by Choi et al. [22]. Catechin was used as a standard antioxidant for comparison. Compound at different concentrations was added to a methanolic solution of DPPH (0.135 mM) and vortexed and left for 30 minutes in the dark. The absorbance of the reaction mixture was measured spectrophotometrically at 517 nm. DPPH free radical scavenging ability (%) was calculated by using the following formula:

(4) $$\begin{array}{c} \left[\frac{\left(\text{Absorbance of control}-\text{Absorbance of sample}\right)}{\text{Absorbance of control}}\right]\times 100\text{.} \end{array}$$

The result was presented as IC_50_ value which is the concentration required to cause 50% scavenging. The IC_50_ value was calculated from the graph plotted as percent scavenging of DPPH free radical against concentration of each compound.

#### 2.6.2. Hydroxyl Radical Scavenging Assay

The potential of the compounds to scavenge the hydroxyl free radical was assayed by the deoxyribose degradation method as described by Elizabeth and Rao [23]. Catechin was used as a reference antioxidant. Compound at different concentrations was mixed with a reaction mixture containing 2.8 mM 2-deoxy-2-ribose, 20 mM phosphate buffer (pH 7.4), 100 *μ*M FeCl_3_, 100 *μ*M EDTA, 1.0 mM H_2_O_2_, and 100 *μ*M ascorbic acid and incubated at 37°C for 1 hour. This was then followed by the addition of 2.8% trichloro acetic acid (TCA) and 1% TBA. The reaction mixture was heated on a boiling water bath. After cooling, the absorbance was measured at 532 nm against an appropriate blank solution. The result was expressed as percent scavenging of hydroxyl radical in relation to the control. The IC_50_ values were calculated from the graph plotted as percent scavenging of hydroxyl free radical against concentration of each compound.

#### 2.6.3. Reducing Power Assay

The reducing power of the compounds was determined by the method of Oyaizu [24]. The compound (1 ml) was added to a mixture of 0.2 M potassium buffer (2.5 ml) and 1% potassium ferricyanide (2.5 ml) and incubated at 50°C for 20 minutes. Then, 10% TCA solution (2.5 ml) was added to the reaction mixture and centrifuged (3000 rpm) for 10 minutes. Finally, 2.5 ml of solution was mixed with 2.5 ml of double distilled water and 0.5 ml of 0.1% ferric chloride solution. The absorbance of the reaction mixture was recorded at 700 nm. A standard antioxidant catechin was used for comparison.

#### 2.6.4. Total Antioxidant Capacity

The antioxidant capacity of the compounds was assessed by the method as described earlier [25]. The compound was added to a mixture of sulphuric acid (0.6 M), sodium phosphate (28 mM), and ammonium molybdate (4 mM) and heated in a water bath at 95°C for 90 min. After cooling to room temperature, the absorbance of the mixture was recorded at 695 nm against blank. A standard antioxidant catechin was used for comparison.

#### 2.6.5. Lipid Peroxidation Inhibition Assay

The lipid peroxidation inhibitory activity of the compounds was determined by the thiobarbituric acid reacting substances (TBARS) method as described by Liu and Ng [26]. Catechin was used as a reference antioxidant. Mouse brain homogenate was employed as the source of lipid which was prepared by homogenizing the brain in the phosphate buffer followed by centrifugation at 10,000 *g* [10]. Lipid peroxidation was induced by ferric ion plus ascorbic acid. The TBARS produced from the reaction of lipid peroxides with thiobarbituric acid was determined colorimetrically at 532 nm. Results were presented as percentage of lipid peroxidation inhibition in relation to the control. The IC_50_ values were determined from the graph plotted as percent inhibition of lipid peroxidation against concentration of each compound.

### 2.7. Statistical Analysis

All statistical data were analyzed using GraphPad Prism (version 8.0.1). and SPSS (version 21.0) Data were presented as mean ± SD. The statistical significance (*P* value <0.05) between the means was determined using one-way analysis of variance (ANOVA).

## 3. Results

### 3.1. Isolation and Identification of Compounds

**Figure 1:**
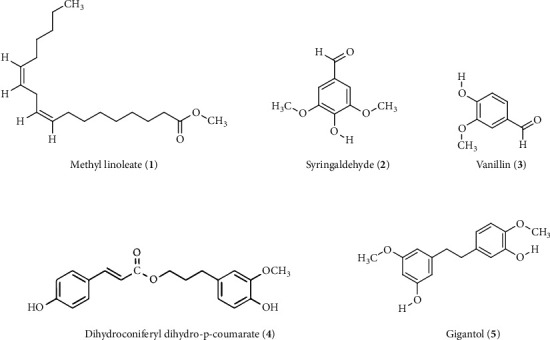
Structures of the isolated compounds 1–5 from the chloroform fraction of *Vanda roxburghii*.

Chromatography of the chloroform fraction, prepared from crude methanol extract of *V. roxburghii* by partitioning, resulted in the isolation of five compounds. The compounds were identified by the comparison of their ^1^H- and ^13^C-NMR spectral data with the published values in the literature [13, 17–19]. The compounds described in Figure 1 included a fatty acid ester: methyl linoleate (1), and four phenolics: syringaldehyde (2), vanillin (3), dihydroconiferyl dihydro-p-coumarate (4), and gigantol (5). All the isolated compounds were investigated for antioxidant and acetylcholinesterase inhibitory potential.

### 3.2. Acetylcholinesterase Inhibitory Activity of the Isolated Compounds

**Figure 2:**
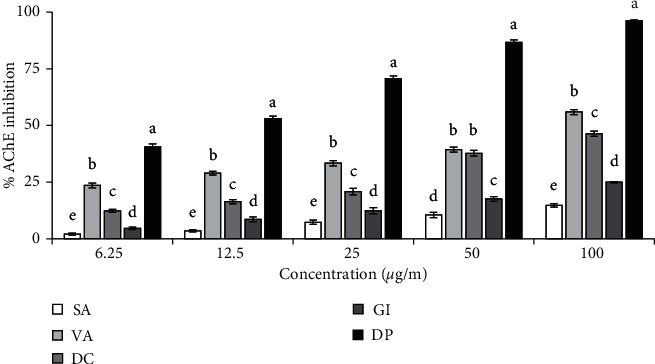
Inhibition of acetylcholinesterase activity by the isolated compounds. IC_50_ (*μ*g/ml): SA, 1268.33 ± 19.14; VA, 84.66 ± 3.20; DC, 118.17 ± 5.06; GI, 547.37 ± 11.47; DP, 10.11 ± 0.26. Results represent mean ± SD (*n* = 3). Donepezil (DP) was used as a reference compound. Compound 1 had no activity and was not shown. Means with different letters (a–e) differ significantly (*P*<0.05). SA, syringaldehyde; VA, vanillin; DC, dihydroconiferyl dihydro-*p*-coumarate; GI, gigantol; DP, donepezil.

The inhibitory potential of the compounds against AChE was evaluated by modified Ellman's method [20], and the result is shown in Figure 2. We determined the IC_50_ values for each compound to assess their potency. Among the compounds, vanillin (3) and dihydroconiferyl dihydro-*p*-coumarate (4) exhibited inhibition of AChE. The IC_50_ values of the compounds were 84.66 ± 3.20 and 118.17 ± 5.06 *μ*g/ml, respectively, indicating that vanillin (3) is more active than dihydroconiferyl dihydro-*p*-coumarate (4). Donepezil was used as the standard AChE inhibitor in this study, whose IC_50_ was found to be 10.11 ± 0.26 *μ*g/ml.

The *Z*-factor of the isolated compounds was determined, and the result is shown in Table S1. The *Z*'-factor of the assay was found to be 0.95, indicating an excellent quality. The *Z*-factor of the compounds was in the range from 0.80 to 0.91, while it was 0.94 for donepezil which was used as positive control. These results clearly indicate the performance of the assay and the compounds.

### 3.3. Antioxidant Potential of the Isolated Compounds

The antioxidant potential of the compounds was evaluated by several in vitro assays in order to understand their free radical scavenging ability, reducing power, total antioxidant capacity and lipid peroxidation inhibitory activity.

#### 3.3.1. DPPH Radical Scavenging Activity

**Figure 3:**
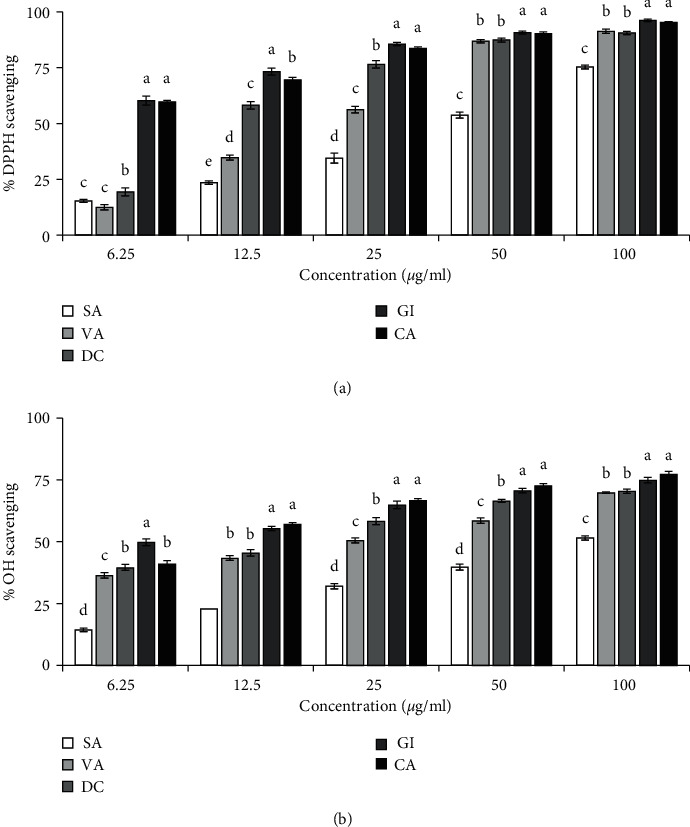
Radical scavenging activity of the isolated compounds. (a) DPPH radical scavenging. IC_50_ (*μ*g/ml): SA, 40.64 ± 1.68; VA, 19.64 ± 0.73; DC, 12.16 ± 0.55; GI, 4.16 ± 0.37; CA, 4.35 ± 0.09. (b) Hydroxyl radical scavenging. IC_50_ (*μ*g/ml): SA, 91.05 ± 3.75; VA, 21.72 ± 0.87; DC, 15.07 ± 1.20; GI, 6.61 ± 0.76; CA, 9.32 ± 0.39. Catechin (CA) was used as a reference antioxidant. Results represent mean ± SD (*n* = 3). Compound 1 had no activity and was not shown. Means with different letters (a–e) differ significantly (*P*<0.05). SA, syringaldehyde; VA, vanillin; DC, dihydroconiferyl dihydro-*p*-coumarate; GI, gigantol; CA, catechin.

DPPH scavenging model is widely used for rapid assessment of antioxidant activity. DPPH is a stable synthetic radical, and upon accepting an electron or hydrogen, it becomes colorless which can be monitored by a spectrophotometer. In this assay, all the compounds except methyl linoleate (1) scavenged the DPPH radical (Figure 3(a)). Among them, gigantol (5) showed the highest activity followed by dihydroconiferyl dihydro-*p*-coumarate (4) and vanillin (3) with IC50 values of 4.16 ± 0.37, 12.16 ± 0.55 and 19.64 ± 0.73 *μ*g/ml. Syringaldehyde (2) was the lowest in their DPPH scavenging potential.

#### 3.3.2. Hydroxyl Radical Scavenging Activity

Hydroxyl radicals are the most toxic free radicals among the radicals produced in the biological system. In the scavenging of hydroxyl radicals generated in vitro by the Fenton reaction, we found that all the five compounds that scavenged DPPH radical also scavenged the hydroxyl radicals (Figure 3(b)). Gigantol (5) was found to be the most potent (IC_50_: 6.61 ± 0.76 *μ*g/ml), and syringaldehyde (2) (IC_50_: 91.05 ± 3.75 *μ*g/ml) was found to be the least potent. The ranking of compounds as judged from the IC_50_ values was gigantol (5) > dihydroconiferyl dihydro-*p*-coumarate (4) > vanillin (3) > syringaldehyde (2).

#### 3.3.3. Reducing Power

**Figure 4:**
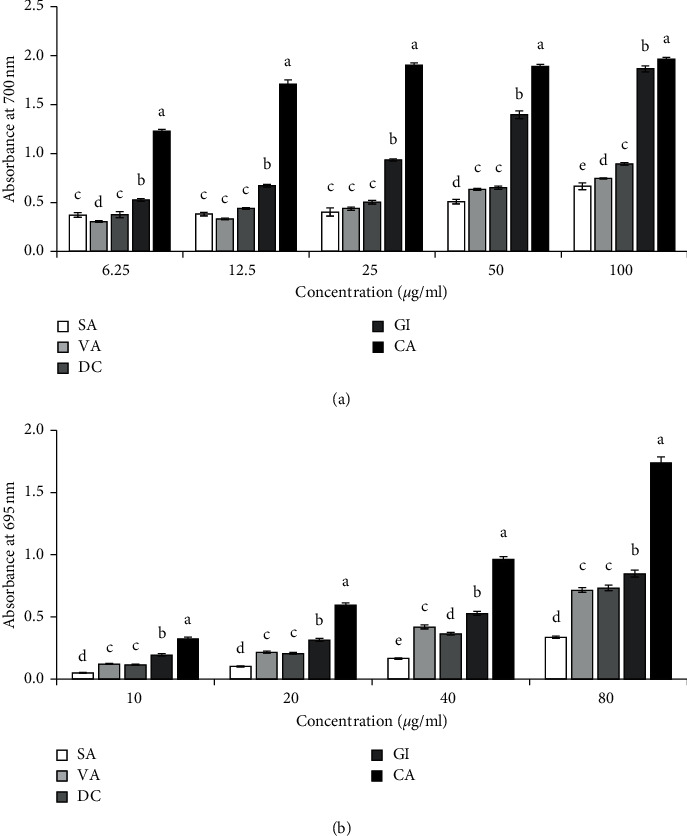
Reducing power and total antioxidant activities of the isolated compounds. (a) Reducing power. Absorbance (100 *μ*g/ml, 700 nm): SA, 0.667 ± 0.035; VA, 0.746 ± 0.005; DC, 0.894 ± 0.014; GI, 1.865 ± 0.030; CA, 1.964 ± 0.019. (b) Total antioxidant activity. Absorbance (80 *μ*g/ml, 695 nm): SA, 0.336 ± 0.009; VA, 0.716 ± 0.020; DC, 0.735 ± 0.022; GI, 0.848 ± 0.029; CA, 1.739 ± 0.049. Catechin (CA) was used as a reference antioxidant. Results represent mean ± SD (*n* = 3). Compound 1 had no activity and was not shown. Means with different letters (a–e) differ significantly (*P*<0.05). SA, syringaldehyde; VA, vanillin; DC, dihydroconiferyl dihydro-*p*-coumarate; GI, gigantol; CA, catechin.

The reducing power of the compounds was determined by their ability to reduce from the Fe^+3^-ferricyanide complex to Fe^+2^ form at different concentrations, and the result is shown in Figure 4. All the compounds showed reducing activity in a concentration-dependent manner (Figure 4(a)). At a high concentration of 80 *μ*g/ml, the absorbance of syringaldehyde (2), vanillin (3), dihydroconiferyl dihydrocoumarate (4), and gigantol (5) was 0.667 ± 0.035, 0.746 ± 0.005, 0.894 ± 0.014, and 1.865 ± 0.030, indicating that gigantol had the highest reducing power followed by coniferyl coumarate, vanillin, and syringaldehyde. These results demonstrated the ability of the compounds to donate electron.

#### 3.3.4. Total Antioxidant Activity

The total antioxidant capacity of the compounds was estimated based on the reduction of Mo (VI) to Mo (V), and the result is presented in Figure 4(b). The result reveals the total antioxidant capacity of all the compounds. Increasing concentration of the compound increased the absorbance which indicates an increase in total antioxidant activity. The absorbance of syringaldehyde (2), vanillin (3), dihydroconiferyl dihydrocoumarate (4), and gigantol (5) at a high concentration of 80 *μ*g/ml was 0.336 ± 0.009, 0.716 ± 0.020, 0.735 ± 0.022, and 0.848 ± 0.029. Therefore, the total antioxidant potential was in the following order: gigantol > coumarate > vanillin > syringaldehyde.

#### 3.3.5. Lipid Peroxidation Inhibitory Activity

**Figure 5:**
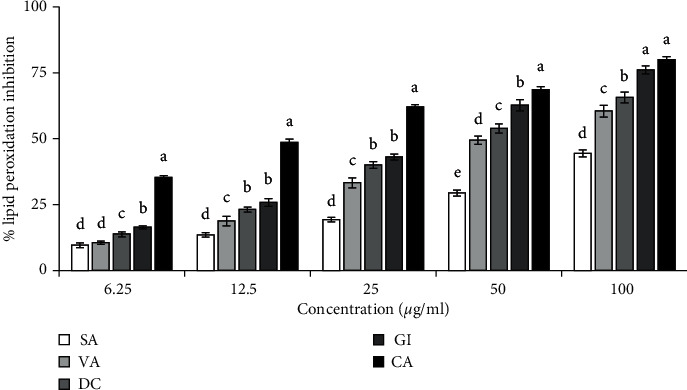
Inhibition of lipid peroxidation by compounds (SA, VA, DC, and GI). IC_50_ (*μ*g/ml): SA, 141.60 ± 6.19; VA, 57.02 ± 2.78; DC, 44.20 ± 1.01; GI, 32.28 ± 1.10; CA, 14.10 ± 0.43. Catechin (CA) was used as a reference antioxidant. Results represent mean ± SD (*n* = 3). Compound 1 had no activity and was not shown. Means with different letters (a–e) differ significantly (*P* < 0.05). SA, syringaldehyde; VA, vanillin; DC, dihydroconiferyl dihydro-*p*-coumarate; GI, gigantol; CA, catechin.

Lipid peroxidation is the consequences of free radical attack on the polyunsaturated fatty acid of the cell membrane. In the inhibition of brain lipid peroxidation induced by ferric ion-ascorbate system, gigantol (5) showed the highest activity followed by dihydroconiferyl dihydro-*p*-coumarate (4), vanillin (3), and syringaldehyde (2) with IC_50_ values of 32.28 ± 1.10, 44.20 ± 1.01, 57.02 ± 2.78, and 141.60 ± 6.19 *μ*g/ml, respectively (Figure 5). Taken together, in all assays, the compounds syringaldehyde (2), vanillin (3), dihydroconiferyl dihydro-*p*-coumarate (4), and gigantol (5) showed the antioxidant activity.

## 4. Discussion

Traditional medicine is practiced in many countries to combat diseases such as AD. *V. roxburghii* is a medicinal herb used to treat central nervous system disorders including AD [11]. To rationalize the usage of traditional medicine as well as to discover a lead compound from medicinal plant, it is necessary to identify the active compounds and elucidate their role in biological activity. Many plant-derived compounds have shown promising anti-AD activity and are currently used in the treatment of AD [10]. In an earlier investigation, we reported the acetylcholinesterase inhibitory and antioxidant properties of *V. roxburghii*, and the highest bioactivities were found in the chloroform fraction [13]. In this study, we have identified four compounds: a fatty acid ester: methyl linoleate (1), and three phenolics: syringaldehyde (2), vanillin (3), and dihydroconiferyl dihydro-*p*-coumarate (4) along with the previously isolated gigantol (5) from the chloroform fraction of *V. roxburghii* (Figure 1). We report for the first time the isolation of these compounds from this species as well as the genus Vanda; they have been found earlier in *Dendrobium* genus of the same Loranthaceae family [19].

Inhibitors of AChE are currently considered as the first line pharmacotherapeutics for the treatment of AD. In addition, antioxidants have also been suggested in AD to prevent the oxidative stress linked to the pathogenesis [10]. We have evaluated and compared the antioxidant and AChE inhibitory activities of the five compounds. Among the isolates, vanillin and dihydroconiferyl dihydro-*p*-coumarate were found to significantly inhibit the activity of AChE, scavenge the free radicals, exhibit the reducing activity and total antioxidant activity, and reduce the peroxidation of lipids (Figures 1–5). The potential of vanillin and dihydroconiferyl dihydro-*p*-coumarate in the inhibition of acetylcholinesterase suggests that they might be responsible for the activity of the chloroform fraction. The AChE inhibitory and antioxidant activity of vanillin has been reported earlier by Kundu and Mitra [27] and Harish et al. [28], which is consistent with our result. Vanillin, a naturally occurring flavoring agent and a major phenolic constituent of the vanilla beans has been described as a multifunctional agent with the ability to inhibit the activity of acetylcholinesterase, reduce the production of free radicals, and decrease the production of nitric oxide and proinflammatory cytokines [27–30]. The compound has shown significant improvement in cognitive impairment in mice induced by scopolamine [31]. Dihydroconiferyl dihydro-*p*-coumarate, a common phenolic constituent of the orchid plants, has been isolated first in 1993 from *Ephemerantha fimbriata* [32], but since then no biological activity of this compound has been known so far. We report for the first time that dihydroconiferyl dihydro-*p*-coumarate is a potent antioxidant and exhibits substantial AChE inhibitory activity (Figures 1–5). Gigantol and syringaldehyde, despite lacking the activity against AChE, were found to exert antioxidant activity (Figures 2–5). These results were in accordance with the results reported earlier [13, 33, 34]. Importantly, gigantol was found to possess the highest antioxidant activity among the compounds in terms of free radical scavenging, reducing power, total antioxidant activity, and inhibition of lipid peroxidation. This suggests that gigantol plays a major in the antioxidant acivity of the chloroform fraction. Syringaldehyde is a phenolic constituent found in many plants and is well known for its antioxidant and anti-inflammatory activity [35]. Recently, this compound has shown neuroprotective effects against cell damage in the brain through anti-oxidative and anti-apoptosis properties in a rat model [36]. However, the potential activity of the identified compounds suggests that these compounds are attributed to the bioactivity of the chloroform fraction and support the traditional use of *V. roxburghii* in the management of AD. Extensive studies with these bioactive compounds in animal model may contribute to the development of high-value phytomedicinal preparation in the treatment of AD.

## 5. Conclusion

In conclusion, four compounds including three phenolics were isolated and identified from the chloroform fraction of *Vanda roxburghii* extract that are contributing to the acetylcholinesterase inhibitory and antioxidant activities. To the best of our knowledge, this is the first report of isolation of these compounds from this plant as well as from the genus Vanda. Further studies in animal model of AD are needed to confirm the therapeutic potential of the extract and compounds from *V. roxburghii*.

## Data Availability

The datasets used and/or analyzed during the current study are available from the corresponding author upon request.

## Supplementary Materials

Supplementary Materials Supplementary Table S1: the *Z*-factor of acetylcholinesterase inhibitory activity of the compounds and standard.
