# Supplementary material for: Acetylcholinesterase Inhibitory and Antioxidant Activity of the Compounds Isolated from Vanda roxburghii
Source: Adv Pharmacol Pharm Sci. 2021 Mar 27;2021:5569054. doi: 10.1155/2021/5569054 (PMC8019628; doi:10.1155/2021/5569054)
Supplement: Supplementary Materials — Supplementary Table S1: the Z-factor of acetylcholinesterase inhibitory activity of the compounds and standard. [file 5569054.f1.docx]

**Table S1.** The Z-factor of acetylcholinesterase inhibitory activity of the compounds and standard.

| Compounds | Z value |
| --- | --- |
| 2  3  4  5  Standard | 0.80  0.91  0.87  0.80  0.94 |
